# Lipopolysaccharide induced acute inflammation leads to a higher systemic and brain exposure to olanzapine in mice

**DOI:** 10.1192/j.eurpsy.2025.758

**Published:** 2025-08-26

**Authors:** J. Hubenak, J. Chladek, J. Masopust, M. Mzik, D. Bayer, C. Andrys, S. Micuda

**Affiliations:** 1 Department of psychiatry, University Hospital Hradec Kralove; 2 Faculty of medicine in Hradec Kralove; 3 Faculty of medicine in Hradec Kralove, Department of Pharmacology, Charles University; 4 Department of clinical biochemistry and diagnostics; 5 Department of clinical immunology and allergology, University Hospital Hradec Kralove, Hradec Kralove, Czech Republic

## Abstract

**Introduction:**

Pro-inflammatory mediators inhibit drug metabolism and transport. Detailed knowledge is lacking on the mechanism and extent of alterations in olanzapine pharmacokinetics during acute inflammatory states accompanying infections.

**Objectives:**

To quantify the extent of systemic and brain exposure to olanzapine in a murine model of endotoxemia compared to a non-endotoxemia model.

**Methods:**

Acute endotoxemia model was established in C57BL/6N mice intraperitonealy injected with 5 mg/kg *Escherichia coli* lipopolysaccharide (LPS). On Day 2 following LPS administration, LPS-injected mice and saline-treated controls were given single doses of olanzapine orally (p.o.) or intravenously (i.v.) or desmethylolanzapine (DMO) i.v. Concentrations and unbound fractions of olanzapine and DMO were measured in plasma samples and brain homogenates. Moreover, plasma biochemistry parameters and mRNA expression patterns were evaluated of pro-inflammatory cytokines, selected phase I and II drug-metabolizing enzymes and transporters in the liver, ileum and brain.

**Results:**

Following p.o. olanzapine, the areas under the concentration-time curve (AUC) for olanzapine and DMO in the plasma were increased 3.8-fold and 2.6-fold (P<0.05) in LPS-injected mice vs. controls. The AUC for olanzapine in the brain homogenate was 5.2-fold higher (P<0.05). Brain DMO was hardly detectable in both groups. The penetration ratios (K_p,brain_) of 8.5 and 6.3 confirmed that LPS increased the passage of olanzapine into the brain. Expression of mRNAs was decreased in the liver of CYP1A2 and UGT1a1/1a5 enzymes and Abcb1a, Bsep and Ntcp transporters and of ileal Abcb1a, whereas Abcb1a and Abcb1b in the brain and inflammatory cytokines and chemokines mRNAs in the liver were upregulated.

**Conclusions:**

Investigation of olanzapine pharmacokinetics in endotoxemia mice clearly indicates a considerable increase in systemic and brain concentrations of the drug after oral administration. Further studies should clarify whether or not the inflammation-induced inhibition of metabolism and efflux transport results in brain overexposure to the drug and adverse effects in accutely infected patients treated with oral olanzapine.

**Disclosure of Interest:**

None Declared

